# Effects of Herb-Partitioned Moxibustion on the miRNA Expression Profiles in Colon from Rats with DSS-Induced Ulcerative Colitis

**DOI:** 10.1155/2017/1767301

**Published:** 2017-01-26

**Authors:** Yan Huang, Zhe Ma, Yun-hua Cui, Hong-sheng Dong, Ji-meng Zhao, Chuan-zi Dou, Hui-rong Liu, Jing Li, Huan-gan Wu

**Affiliations:** ^1^Key Laboratory of Acupuncture and Immunological Effects, Shanghai University of Traditional Chinese Medicine, Shanghai 200030, China; ^2^Shanghai Research Institute of Acupuncture and Meridian, Shanghai University of Traditional Chinese Medicine, Shanghai 200030, China; ^3^Yueyang Clinical Medical College, Shanghai University of Traditional Chinese Medicine, Shanghai 200437, China; ^4^Department of Acupuncture and Moxibustion, Shanghai Yueyang Hospital of Integrated Traditional Chinese and Western Medicine, Shanghai 200437, China

## Abstract

*Objective.* This study explored the mechanism of herb-partitioned moxibustion (HM) on dextran sulfate sodium- (DSS-) induced ulcerative colitis (UC) from the miRNA perspective.* Methods.* Rats were randomly divided into 3 groups [normal control (NC) group, UC model (UC) group, and herb-partitioned moxibustion (UCHM) group]. The UC and UCHM groups were administered 4% DSS for 7 days. The UCHM group received HM at the Tianshu (bilateral, ST25). The effect of HM on UC was observed and the miRNA expression profile in the colon tissues was analyzed.* Results.* Compared with the UC group, the body weights were significantly higher in the UCHM group on day 14 (*P* < 0.001); the macroscopic colon injury scores and microscopic histopathology scores in the UCHM group decreased (*P* < 0.05); and there were 15 differentially expressed miRNAs in the UCHM group. The changes in miR-184 and miR-490-5p expression levels on the UC were reversed by HM intervention. Validation using qRT-PCR showed that two miRNAs expression trend was consistent with the sequencing results.* Conclusion.* HM at ST25 might regulate miR-184 and miR-490-5p expression, act on the transcription of their target genes to regulate inflammatory signaling pathways, and attenuate inflammation and tissue injury in the colons of rats with DSS-induced UC.

## 1. Introduction

Ulcerative colitis (UC) is a group of chronic, nonspecific, intestinal inflammatory diseases with unclear etiology. The incidence of UC gradually increases annually in response to changes in lifestyle, dietary structure, and living conditions [1, 2]. According to literature reports, 3.7–5.7% of UC patients have colorectal cancer due to long-term and extensive colonic ulcers and inflammation [3]. Early intervention for UC can prevent carcinogenesis. The pathogenic factors underlying UC are complicated; therefore, genetic and environmental factors have received increasing attention. However, the pathogenic mechanism is not completely clear, which makes treatment difficult. UC has a long disease course and is prone to recurrence; currently, there is no effective radical measure, and long-term drug treatment is required. Furthermore, the long-term safety of biological products requires confirmation in future studies, and the expensive prices and potential side effects limit their clinical application to some extent [4]. Therefore, in-depth studies on the pathogenic mechanism of UC are active for both diagnosis and treatment.

Recently, the association between noncoding RNAs and inflammation has been closely investigated. MicroRNAs (miRNAs) are small noncoding single-stranded RNAs that are 18–25 nucleotides in length. They regulate gene expression through binding to the 3′-untranslated region (UTR) of mRNAs encoding target proteins to play an epigenetic posttranscriptional role. The biological functions of miRNAs are associated with adaption to physiological and pathological environments and the reversal or change of gene expression in completely differentiated tissues [5, 6]. miRNAs are the basic regulatory method of inflammation-type signaling pathways and can cause different inflammatory diseases [7, 8], including inflammatory bowel disease (IBD) [9, 10]. Recent studies have identified dysfunctional miRNAs in the peripheral blood and colon tissues in UC diseases [11–14]. Regulation of miRNA expression levels can inhibit the inflammatory reaction in the colon, which has potential for UC treatment and the prevention of precancerous lesions [15].

Joos et al. performed a randomized controlled study to show that acupuncture and moxibustion of traditional Chinese medicine (TCM) could improve the quality of life of UC patients [16]. Over the last 30 years, our group has concentrated on the study of the effect of moxibustion (a characteristic treatment in TCM) on UC. The combined use of herb-partitioned moxibustion (HM) and the unrestricted use of Western medicine by patients can correct pathological changes of the mucosa in the colon and effectively control intestinal inflammation [17–19]. However, the mechanism underlying the effect of HM on UC is not completely clear. HM might change miRNA expression levels in the colons of UC rats to inhibit inflammatory functions. We used the UC rat model induced by 4% dextran sulfate sodium (DSS) and applied HM on bilateral ST25 points to observe the intervention effect. Thus, for the first time, we studied the regulatory function of moxibustion on miRNAs in the colons of UC rats from a miRNA perspective.

## 2. Materials and Methods

### 2.1. Main Equipment and Reagents

The NanoDrop-2000 spectrophotometer was supplied by NanoDrop Technologies (Wilmington, DE, USA). The TRIzol reagent was supplied by Invitrogen (Life Technologies, Carlsbad, CA, USA). The TapeStation 2200 (Agilent Technologies Inc., Santa Clara, CA, USA), Illumina TruSeq™ Small RNA Sample Prep Kit (Illumina, San Diego, CA, USA), Qubit® 2.0 Fluorometer using the dsDNA HS and/or BR assay kit (Life Technologies, Carlsbad, CA, USA), Illumina HiSeq 2000 sequencer (Illumina, San Diego, CA, USA), 36–50 kDa DSS (#160110, MP Biomedicals, CA, USA), Bio-Rad CFX96TM Real-Time PCR system (Bio-Rad Laboratories, CA, USA), TruSeq PE Cluster Kit v3-cBot-HS (Illumina, San Diego, CA, USA), RNase inhibitor (Thermo Fisher Scientific, Rockford, IL, USA), and iQTM SYBR® Green Supermix (Bio-Rad, Hercules, CA, USA) were obtained from the indicated manufacturers.

### 2.2. Animals

Healthy adult male Sprague-Dawley (SD) rats with body weights of 180 ± 20 g were provided by the Experimental Animal Center of Shanghai University of Traditional Chinese Medicine and were purchased from Vital River Laboratory Animal Technology Co. Ltd. (Beijing, China). The license for the use of experimental animals was SCXK (Beijing) 2012-0001. The housing conditions were clean-grade, 12 h of alternating day and night (illumination from 7 a.m.–7 p.m.), 20 ± 2°C room temperature, and 50–70% indoor humidity. After 1 week of adaptive feeding, the formal experiments began. The experiments were performed in strict accordance with the guidelines for experimental animals of the National Institute of Health (NIH) of the USA. Animal treatment during the experimental process followed the regulations of “Instructive Notions with respect to Caring for Laboratory Animals” released by the Ministry of Science and Technology, China.

### 2.3. Establishment of the UC Model

**Figure 1:**
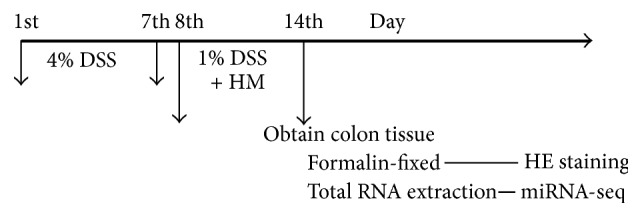
Flowchart. The model was established using 4% DSS between days 1 and 7 and maintained using 1% DSS between days 8 and 14. Herb-partitioned moxibustion (HM) was applied at the Tianshu points (bilateral, ST25).

After 1 week of adaptive feeding, the formal experiments began. The 36 rats were randomly divided into the following 3 groups: normal control (NC) group, UC model (UC) group, and herb-partitioned moxibustion (UCHM) group. Each group had 12 animals. The UC and UCHM groups both received 4% DSS in water ad libitum for 7 days. After model establishment, two animals from each group were selected for hematoxylin and eosin (HE) staining to determine whether the model was successfully established. After the model was successfully established, the animals were provided 1% DSS in water ad libitum for 7 days. The UCHM group was also provided with the moxibustion intervention. The body weights of the rats in each group were measured on the first day of the intervention (Figure 1).

### 2.4. Moxibustion Intervention

**Figure 2:**
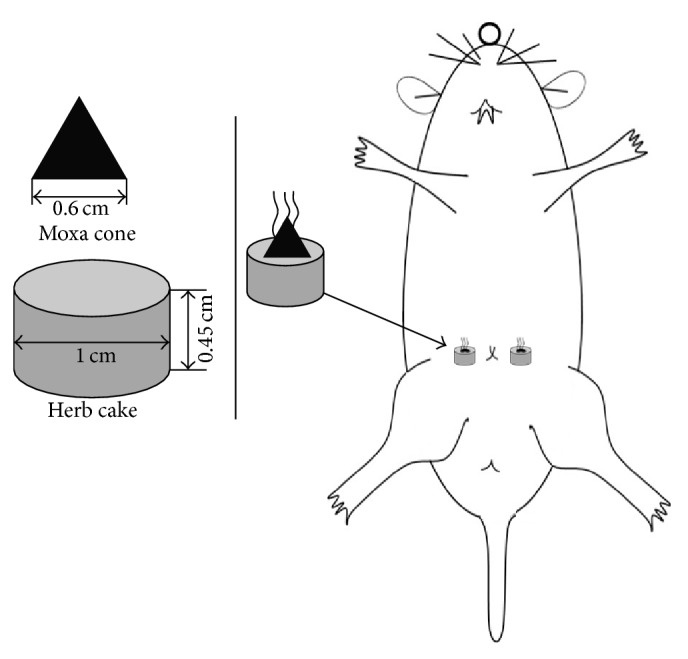
Schematic diagram of HM at the rat ST25 points (bilateral).

UCHM group: the Tianshu (bilateral, ST25) points were selected. The medicinal cake was Chinese medicine powder (*Coptis chinensis*,* Radix aconiti lateralis, Cortex Cinnamomi*,* Radix aucklandiae*,* Flos carthami*,* Salvia miltiorrhiza*, and* Angelica sinensis*) mixed and stirred with yellow wine to form a thick paste. The medicinal cake was prepared with 1 cm and 0.45 thicknesses using a mold. The moxa cone was prepared with a 0.6 cm diameter and height using a mold; the weight was 90 mg. The rats were immobilized using a fixator, and the ST25 points (bilateral) were exposed. Rat hair at the ST25 points was shaved one day in advance. The prepared moxa cone was placed on the top of a medicinal cake, and the medicinal cake was placed at the ST25 points (bilateral). The moxa floss was lit; each acupoint used 2 moxa cones per time point. Moxibustion was performed once every day in the morning for intervention for 7 days (Figure 2). Rats from the NC and UC groups were immobilized using the same method applied to the UCHM group. The immobilization time was the same as that in the UCHM group.

### 2.5. Sample Collection

After 7 days of HM intervention, all of the rats were anesthetized using 2% pentobarbital sodium (30–40 mg/kg). The distal colon at a length of 6–8 cm was collected 1 cm from the anus. Each colon was divided into 2 parts. One part was fixed in 4% paraformaldehyde. The other part was minced and placed in a cryotube, frozen in liquid nitrogen for 1 h, and stored in a −80°C freezer for future use.

### 2.6. Macroscopic Scoring of Colon Injury

The collected distal colon was cut open along the mesenterium and spread flat. Adhesion and bleeding of the mucosa were observed. The scoring of the distal colon of the rats was performed according to the Score Criteria of Colonic General Damage [20, 21].

### 2.7. Observation of Histopathology and Microscopic Scoring of the Colon

Colon tissues fixed in paraformaldehyde for 24 h were removed, cleaned, trimmed, dehydrated, embedded, and sectioned into 4 *μ*m thin sections. Histopathological changes of the colon were observed using HE staining under a light microscope. Microscopic scoring of the colon was performed according to the Score Criteria of Colonic Histological Damage [22].

### 2.8. Extraction and Quality Control of Total RNA and Construction of the cDNA Library of Small RNAs

Two colon tissues were collected from each group. Total RNA was extracted from the colon tissues using the TRIzol method. The purity and concentration were detected using the NanoDrop 2000. The RNA integrity was detected using the Agilent 2200. Total RNA samples with RIN (RNA integrity number) values higher than 8 were used for library construction. A total of 3 *μ*g of total RNA from each sample was used to construct the small RNA library. Different index tags were selected for library construction based on the operational manual of the TruSeq Small RNA Sample Prep Kit (Illumina, San Diego, CA, USA). The quality of the library after construction was examined using the Agilent 2200.

### 2.9. Small RNA-Seq and Data Analysis

This experiment used the TruSeq PE Cluster Kit v3-cBot-HS (Illumina) reagent to generate clusters on cBOT. Next, the single-terminal sequencing program was performed on the HiSeq 2000 sequencing platform. The data collection software provided by Illumina was used to control the sequencing process and real-time data analysis. The results were compared with the reference sequences, and DEseq was used for the analysis. The fold changes and *P* values were calculated. FC ≥ 1.5 was set as upregulation and ≤0.5 as downregulation (*P* value < 0.05).

### 2.10. miRNA Target Gene Prediction

The miRNA target gene prediction was performed using a combination of MIRanda (http://www.microrna.org/microrna/home.do), miRDB (http://mirdb.org/miRDB/), and TargetScan6.2 (http://www.targetscan.org/vert_60/).

### 2.11. miRNA Target Gene Pathway and GO Analysis

Signaling pathways with significant enrichment of target genes were analyzed using http://prediction.ebioservice.com:8080/path/. Additionally, Gene Ontology (GO) enrichment analysis was performed, with a *P* value < 0.05 indicating significant enrichment.

### 2.12. Validation of miRNAs Using qRT-PCR

The samples were amplified, and 2 miRNAs (miR-184 and miR-490-5p) were validated using quantitative real-time PCR. U6, miR-184, and miR-490-5p were synthesized and purified by RiboBio (Guangzhou, China). The 2^−ΔΔCt^ method was performed to analyze relative miRNA expression in 5 replicate samples. The one-way analysis of variance (ANOVA) method was performed to assess the significance of differences in the relative miRNA expression levels between the 3 groups (*P* < 0.05).

### 2.13. Statistical Analysis

The SPSS 22.0 software was used for the analysis. The normality test and the homogeneity of variance test were performed on the data. The data were presented as the mean ± standard deviation $\left(\overset{-}{x}\pm s\right)$ if they conformed to the normal distribution. Data that did not conform to the normal distribution were presented as the median (P_25_, P_75_). If the data conformed to the normal distribution, the comparison of differences among groups was performed using one-way ANOVA. If the variances were homogeneous, the pairwise comparison was performed using the least significant difference (LSD) test. If the variances of all groups were not homogenous, the nonparametric Kruskal-Wallis *H* test was performed for the analysis. The pairwise comparison was performed using the Nemenyi method. If the data did not conform to the normal distribution, the rank sum test was performed. The significance level of the statistical examination was *α* = 0.05. *P* < 0.05 indicated that the difference had statistical significance.

## 3. Results

### 3.1. HM at the ST25 Points Attenuated DSS-Induced UC

**Figure 3:**
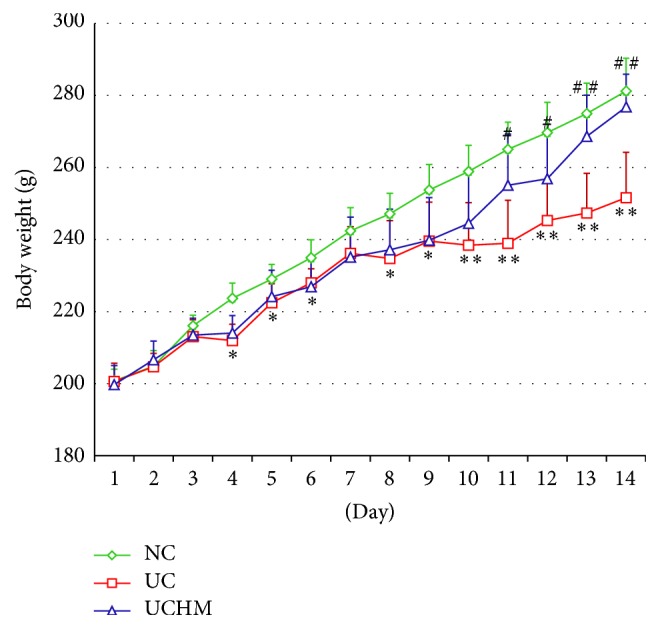
Comparison of body weights of rats in all groups at different time points. Compared to the NC group, the body weights of rats in the UC group decreased (^*∗*^*P* < 0.05, ^*∗∗*^*P* < 0.001). Compared to the UC group, the body weights of rats in the UCHM group increased (^#^*P* < 0.05, ^##^*P* < 0.001). NC: normal group; UC, UC group; UCHM, herb-partitioned moxibustion group (each group, *n* = 10).

The body weights of the rats with DSS-induced UC significantly changed. The body weights of rats in the UC group decreased on days 4–8 (*P* < 0.05; except for day 7) and significantly decreased on days 10–14 (*P* < 0.001) compared to the NC group rats. The body weights of rats in the UCHM group increased on days 11-12 (*P* < 0.05) and significantly increased on days 13-14 (*P* < 0.001) compared to the UC group rats (Figure 3).

**Figure 4:**
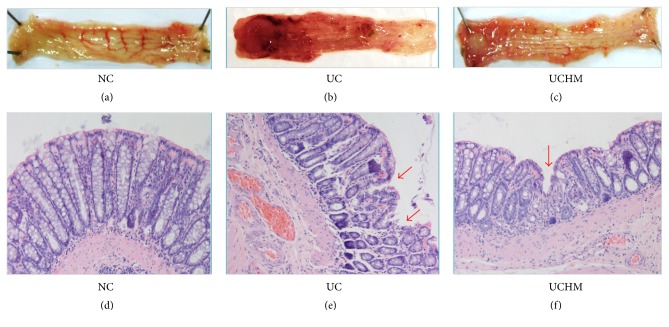
Colon tissue injuries in all groups. (a) and (d) were both NC groups. (b) and (e) were both UC groups. (c) and (f) were both UCHM groups. (a), (b), (c) represent the changes of the representative colon observed by the naked eye. (d), (e), (f) show representative HE staining (HE, ×200) of the histopathological changes of colon tissues under light microscopy.

**Figure 5:**
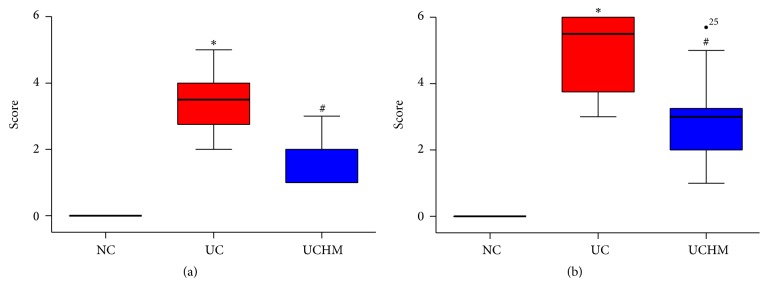
Comparison of colon tissue injury scores among all groups. Macroscopic score; (b) histopathological score. NC, normal group; UC, UC group; UCHM, herb-partitioned moxibustion group (each group, *n* = 10). Compared with the NC group, ^*∗*^*P* < 0.05; compared with the UC group, ^#^*P* < 0.05.

The rats with DSS-induced UC had loose stools, bloody stools, decreased activities, and slow response times. Gross injury of the rat colon was observed during collection. In the NC group, the colon did not exhibit adhesion or bleeding points; the inner wall of the colon was clean and smooth. In the UC group, the colon exhibited significant adhesion and multiple bleeding points; the inner wall was not smooth, and residual muck, such as blood and loose stool, was observed. In the UCHM group, the adhesion was attenuated compared to the UC group, and the bleeding points were decreased, the inner wall was not smooth, and a small amount of or no residual blood and loose stool were observed (Figures 4(a)–4(c)). Statistical analysis of the gross injury scores showed that the macroscopic colon injury scores in the UC group were significantly increased compared to the scores in the NC group (*P* < 0.05). The macroscopic colon injury scores in the UCHM group were significantly decreased compared to the scores in the UC group (*P* < 0.05) (Figure 5(a)).

After the observations of gross injury, microscopic changes of the histopathology of the colon tissues after HE staining were evaluated using light microscopy. In the NC group, the structures of the intestinal mucosa and glands were intact, and no congestion, edema, or necrosis was observed. In the UC group, the intestinal mucosa was not intact, the gland structure was destroyed, and the morphology was different; we observed ulceration, the mucosa and submucosa exhibited moderate congestion and edema, and there was a large amount of neutrophil, lymphocyte, and plasma cell infiltration. In the UCHM group, ulcer healing and epithelial hyperplasia covering the mucosa on the surface of the ulcer were observed; additionally, the morphology of the peripheral glands was irregular, the mucosa and submucosa had mild congestion and edema, and there was a small amount of lymphocyte and plasma cell infiltration (Figures 4(d)–4(f)). Statistical analysis of the microscopic change scores in the histopathology of the colon tissues showed an increase in rats in the UC group compared to rats in the NC group (*P* < 0.05). Conversely, the microscopic scores of colon tissues in the UCHM group were decreased compared to the rats in the UC group (*P* < 0.05) (Figure 5(b)).

Through the above observations of body weight, gross colon tissue injury, and histopathology, we found that HM had better intervention effects on DSS-induced UC and could correct histopathological changes of the colon, attenuate intestinal inflammation, and increase the body weight of the rats. Next, colon tissues were collected from two rats per group for total RNA extraction. Small RNA sequencing technology was used to observe the effect of HM on the miRNA expression profiles in the colon tissues of rats with DSS-induced UC.

### 3.2. Differentially Expressed miRNAs in Colon Tissues with DSS-Induced UC

**Figure 6:**
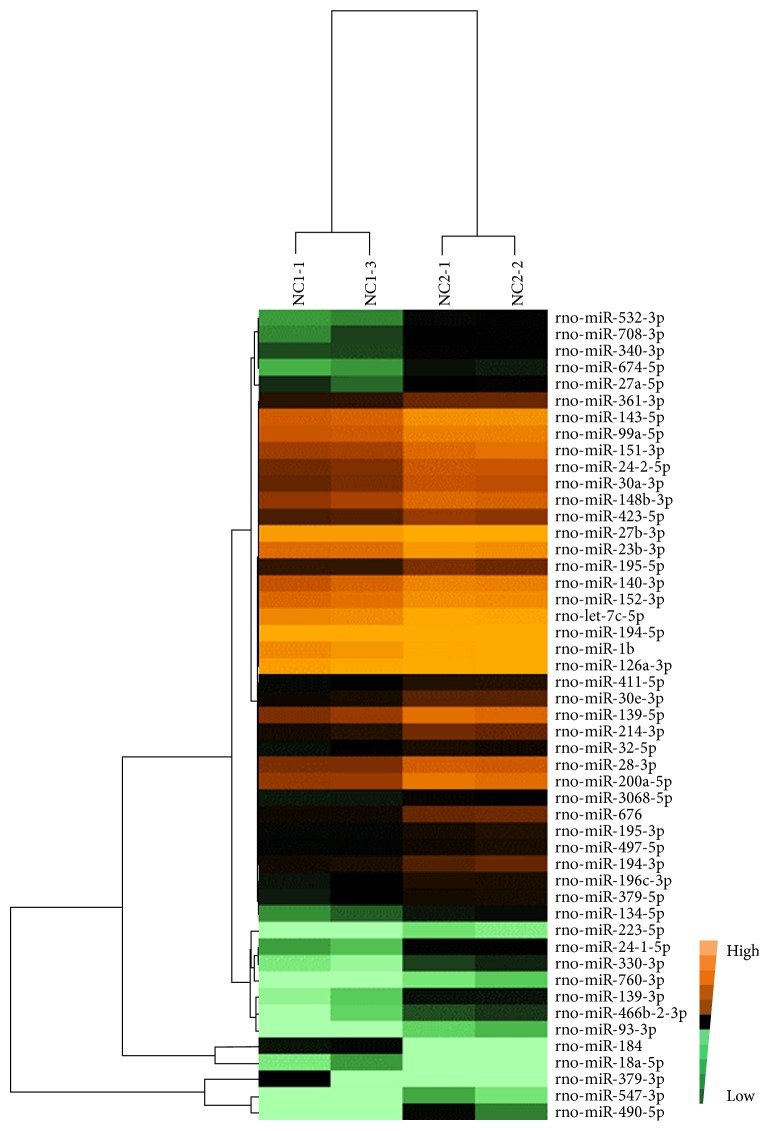
The differentially expressed miRNA profile of UC versus NC. The bright brown color indicates the highest expression. The expression decreases with the darkening of the brown color. The black color indicates that the expression is 0. A change in color from bright green to dark green indicates a gradual decrease in expression.

Through bioinformatics analysis, miRNAs with fold change ratios larger than 1.5 in each group were defined as upregulated miRNAs, and miRNAs with fold change ratios less than 0.5 were defined as downregulated miRNAs; *P* values smaller than 0.05 indicated significant differences. Expression values lower than 0.001 were filtered. The heat map of the differentially expressed miRNAs was analyzed using the Cluster 3.0 and Treeview software to intuitively assess the homogeneity in each sample and the expression differences among all of the groups. The heat map showed that the expression of two samples in each group was more consistent and that there were significant differences between two groups. There were 49 differentially expressed miRNAs in the colon tissues of the UC group compared to the NC group, of which 47 were upregulated and 2 were downregulated; miR-184 and miR-18a-5p were both downregulated (Figure 6). There were 8 miRNAs with fold changes in expression greater than 3 [miR-490-5p (FC = 9.44), miR-547-3p (FC = 4.77), miR-24-1-5p (FC = 3.78), miR-200a-5p (FC = 3.55), miR-139-3p (FC = 3.51), miR-139-5p (FC = 3.46), miR-676 (FC = 3.32), and miR-532-3p (FC = 3.07)]. Among them, miRNA-490-5p was upregulated 9.44-fold, which represented the largest change.

### 3.3. Changes in Differentially Expressed miRNAs in Colon Tissues from Rats with DSS-Induced UC in Response to HM at the ST25 Points

**Figure 7:**
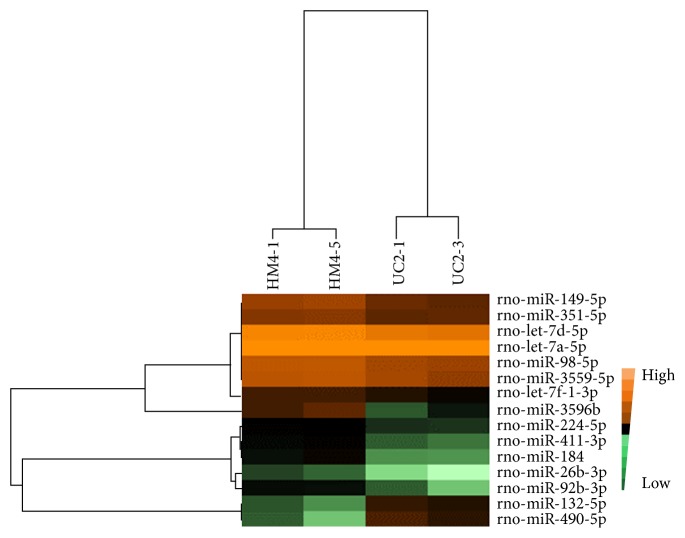
The differentially expressed miRNA profile of UCHM versus UC. The bright brown color indicates the highest expression. The expression decreases with the darkening of the brown color. The black color indicates that the expression was 0. A change in color from bright green to dark green color indicates a gradual decrease in expression.

The heat map showed that the expression of two samples in each group was more consistent and that there were significant differences between two groups. There were 15 differentially expressed miRNAs in the colon tissues of the UCHM group compared to the UC group, of which 13 were upregulated and 2 were downregulated. The upregulated miRNAs in the colon tissues of UC rats changed by HM were miR-149-5p, miR-351-5p, let-7d-5p, miR-98-5p, let-7a-5p, miR-3559-5p, let-7f-1-3p, miR-3596b, miR-224-5p, miR-411-3p, miR-184, miR-26b-3p, and miR-92b-3p. The downregulated miRNAs were miR-132-5p and miR-490-5p (Figure 7). There was only 1 miRNA with a fold change of expression higher than 3 (miR-3596b; FC = 4.14); this miRNA exhibited the largest change.

There were two common differentially expressed miRNAs in the two heat maps shown in Figures 6 and 7 (miR-184 and miR-490-5p). miR-184 was downregulated by the UC pathological status; after the HM intervention, its expression was upregulated. While miR-490-5p was upregulated by the UC pathological status, its expression was downregulated after the HM intervention.

### 3.4. Prediction of miRNA Target Genes Coexpressed in Colon Tissues of UC Rats and Regulated by HM

**Figure 8:**
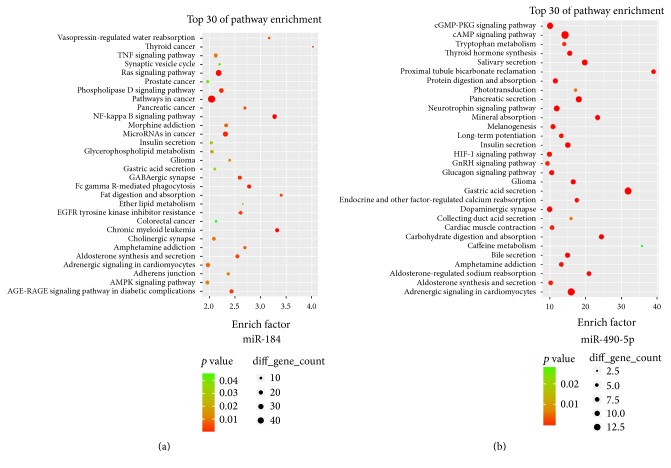
Analysis of pathways related to the predicted miRNA target genes. Pathway items with the top 30 enrichment levels. The enrichment factor indicated the ratio between the number of differentially expressed genes in this pathway item and the total number of all annotated genes in this pathway item. When the enrichment factor was larger, the level of enrichment was higher. The *P* values ranged between 0 and 0.05. When the value was closer to 0, the enrichment was more significant.

HM altered the expression of 15 miRNAs, of which 2 were associated with UC pathological changes (miR-184 and miR-490-5p). The target genes of rat miRNAs can be predicted using 3 databases (miRanda, miRDB, and Targetscan). miRNA-184 had 1130 target genes. The relevant top 30 pathways that were closely associated with the occurrence and development of intestinal inflammation and malignant tendencies in UC included the TNF signaling pathway, Ras signaling pathway, pathway in cancer, NF-*κ*B signaling pathway, miRNAs in cancer, colorectal cancer, and AMPK signaling pathway (Figure 8(a)). miR-490-5p had 100 target genes. The relevant top 30 pathways associated with UC included the cGMP-PKG signaling pathway, cAMP signaling pathway, neurotrophin signaling pathway, HIF-1 signaling pathway, and endocrine and other factor-regulated calcium reabsorption (Figure 8(b)).

### 3.5. Sample Amplification and Validation of miRNA Expression in Colon Tissues of All Groups Using qRT-PCR

**Figure 9:**
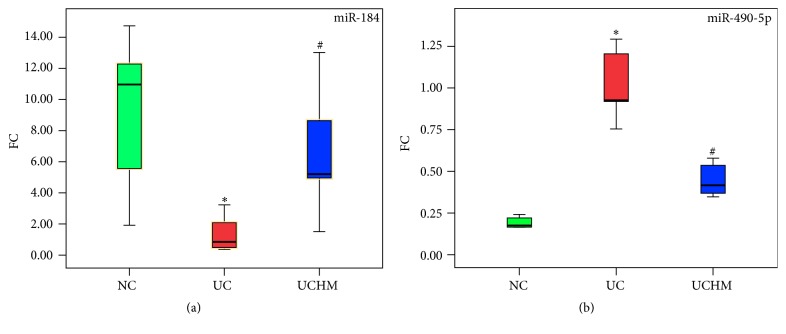
miR-184 and miR-490-5p expression in colon tissues of all groups. (a) miR-184 expression. (b) miR-490-5p expression, NC, normal group; UC: UC group; UCHM, herb-partitioned moxibustion group (each group, *n* = 5). Compared with the NC group, ^*∗*^*P* < 0.05; compared with the UC group, ^#^*P* < 0.05.

The expression of the 2 miRNAs that were reversed by HM was validated using the qRT-PCR method. The miR-184 expression level decreased and the miR-490-5p expression level increased in the UC group compared to the NC group (both *P* < 0.05). The miR-184 expression level increased and the miR-490-5p expression level decreased in the UCHM group compared to the UC group (both *P* < 0.05) (Figures 9(a) and 9(b)).

## 4. Discussion

The major symptoms of UC are abdominal pain, diarrhea, and bloody stool. The active period and the remission period usually alternate, and the disease course is very long. Currently, UC is considered to be associated with a variety of factors, including genetics, environment, diet, intestinal flora, and immunity [23]. However, the specific pathogenic mechanism is unclear. Because UC has a long disease course, recurrent attacks, and delayed healing, UC patients are usually frail. Under the guidance of TCM theories, the moxibustion treatment method can be used. As a traditional therapy method, moxibustion has been increasingly recognized and accepted around the world due to its effectiveness. The commonly used moxibustion methods for UC treatment in China include mild-warm moxibustion, herb-partitioned moxibustion (HM), and ginger-partitioned moxibustion. Over the last 30 years, our group has been devoted to studying the effect of moxibustion on inflammatory intestinal diseases and has found that HM can alleviate the abdominal pain, diarrhea, and bloody stool symptoms in UC patients, thereby indicating definite efficacy in UC patients [17, 18]. In this study, we observed that the general condition of rats with DSS-induced UC treated with HM gradually improved, and the increase in body weight was fast, with the body weights on day 14 close to the body weights in the normal group. The general conditions of rats in the UC group were poor. The increase in body weight was slow, with the body weights on day 14 significantly different from the body weights in the NC and UCHM groups. Macroscopic and microscopic scoring of colon tissue injuries showed that the colons of UC rats after model establishment had significant macroscopic and microscopic injuries. After treatment with HM at the ST25 points in the UC rats, macroscopically, the colon injury was mild, and little bleeding and damage were detected. Microscopically, the histopathology of the colon suggested that the ulcer surface was healed; we observed mucosal epithelial hyperplasia and coverage, and there was little lymphocyte and plasma cell infiltration. These results suggested that HM had protective functions in the injured colon in rats with DSS-induced UC. Previous studies by our group demonstrated that HM regulated immune inflammation, cell apoptosis, and transcription factor expression in UC rats to attenuate colon injuries [24–28]. These effects could impact the biological effects of T and B lymphocytes, neutrophils, and macrophages, which are closely associated with the apoptosis of mucosal epithelial cells and inflammation in the colon, to upregulate the overall immune functions in the body [29–32].

With the development of microarrays and high-throughput sequencing, UC disease-associated miRNAs are gradually being elucidated. These miRNAs can regulate inflammatory cell chemotaxis, transcription factor activity, intestinal epithelium barrier functions, and autophagy activity [33–36]. We hypothesized that the immune regulatory functions of moxibustion on UC were regulated by epigenetic modifications, such as noncoding RNAs. Therefore, to clarify the effectiveness of HM on rats with DSS-induced UC from the perspective of the miRNA expression profile, the miRNA-seq high-throughput sequencing method was performed to investigate the miRNA expression profiles associated with histopathological changes in the colons of rats with DSS-induced UC and to screen changes in the differentially expressed miRNAs in the colons of UC rats in response to HM at the ST25 points. We found that 49 miRNAs exhibited changes in the rats in response to the pathological status of DSS-induced UC. Five of these miRNAs were altered in colon tissues or peripheral blood in previous studies (miR-126, miR-214, miR-532, miR-140, and miR-340). Some miRNAs were involved in immune functions in the colon, and some miRNAs participated in carcinogenesis of the colon. In 2008, Wu et al. first reported 11 differentially expressed miRNAs in colon tissues that were closely associated with UC patients in the active period, of which miR-126 expression was upregulated [33]. miR-126 participated in immune inflammation and carcinogenesis processes in the colon [33, 37, 38] and could directly bind to the 3′-UTR region of the target gene I*κ*B to regulate the NF-*κ*B signaling pathway [39]. In colon tissues from UC and ulcerative colitis-associated colorectal cancer (CRC) subjects, miR-214 expression was significantly upregulated to activate inflammatory responses; colon injury could be further aggravated through the feedback loop mediated by PTEN and PDLIM2, and the transcription factors NF-*κ*B and STAT3 could be regulated to influence the IL-6 expression level. In contrast, miR-214 inhibitors reduced colitis in UC patients and tissues with DSS-induced colitis [40]. miR-532 was expressed in DSS-induced UC colon tissues and was upregulated in the peripheral blood of active and inactive UC patients [11]. miR-532 was associated with inflammatory responses in UC. In contrast to miR-532, miR-340 was only upregulated in the peripheral blood of active UC patients; no changes were observed in the peripheral blood of inactive UC patients [11]. miR-340 was upregulated in the DSS-induced UC colon, suggesting that miR-340 was involved in acute colitis. miR-140 was also found to act on the target gene HDAC4 to overcome resistance to antitumor drugs [41]. Finally, miR-140 participated in the immune response in the colons of rats with DSS-induced UC.

The inflammatory reaction present in local tissues after infection and injury in the body is a common and important basic pathological process. miRNAs play important roles in immune regulation in the colon. Acupuncture and moxibustion have immune regulatory and inflammation inhibition functions, and moxibustion can inhibit colitis in UC subjects [42–45]. The results in this study suggested that changes in miR-184 and miR-490-5p expression levels in colon tissues under the UC pathological status were reversed in the UCHM group. miR-184 was directly associated with the level of inflammation, and its expression was significantly downregulated in inflammatory tissues [46, 47]. miR-184 expression was also significantly downregulated in inflammatory tissues from the colons of DSS-induced UC to participate in the regulation of immune inflammation. However, the expression of miR-184 was significantly increased in tumor tissues, and inhibition of miR-184 inhibited tumor growth [48], which was completely different from its function in inflammatory tissues. miR-490-5p was increased in the UC model, and moxibustion could downregulate its expression. However, its function in inflammatory tissues has not been confirmed at present. Currently, studies have reported that miR-490-5p expression is decreased in tumor tissues, whereas overexpression of miR-490-5p can inhibit tumor growth [49, 50]; these results differ from the mechanism of action in our study. Moreover, miR-98 upregulation could be used as a molecular marker for the colon in active UC patients and was associated with the progression of colitis in UC [10]. Our study showed that miR-98 expression showed a decreasing trend in DSS-induced UC colon tissues that could be reversed by HM. HM also upregulated miR-149-5p expression in colon tissues, whereas miR-149-5p functioned on the transcription factor FOXM1 to inhibit the migration of tumor cells in the colon [51]. miR-224, let-7a, and let-7d were downregulated in colon cancer tissues, and upregulation of miR-224 or let-7a attenuated colon cancer growth [52–55]. The expressions of these 3 miRNAs all showed a decreasing trend in our results; however, HM could promote their upregulation. In summary, HM could change the expression of miRNAs associated with tumor growth or prevent carcinogenesis in UC. We performed qRT-PCR to validate the expression of 2 small RNAs using U6 as the internal control. The results suggested that HM could reverse the expression of miR-184 and miR-490-5p, which changed with the UC pathological status. These results suggested that HM attenuated colon injury in rats with DSS-induced UC at least partially via the regulation of small RNA expression.

Target gene predictions for miR-184 and miR-490-5p and the pathway analysis of the target genes of these 2 small RNAs showed that potential target genes of miR-184 were involved in some pathways that were closely associated with UC, such as the TNF signaling pathway, Ras signaling pathway, pathway in cancer, NF-*κ*B signaling pathway, miRNAs in cancer, colorectal cancer, and AMPK signaling pathway. Similarly, the miR-490-5p target genes were involved in pathways associated with UC, including the cGMP-PKG signaling pathway, cAMP signaling pathway, neurotrophin signaling pathway, HIF-1 signaling pathway, and endocrine and other factor-regulated calcium reabsorption. HM regulated small RNA expression. Thus, HM may act on the transcription of their target genes, thereby attenuating intestinal inflammation in rats with DSS-induced UC. In the future, in-depth studies on the 2 small RNAs will be performed to investigate the mechanism of action of HM at the ST25 points on DSS-induced UC.
